# Validation of Four Prognostic Models for Metastatic Posterior Uveal Melanoma in a Danish Cohort

**DOI:** 10.1167/iovs.66.5.38

**Published:** 2025-05-27

**Authors:** Tine Gadegaard Hindso, Camilla Wium Bjerrum, Kristoffer Nissen, Mette Bagger Sjøl, Carsten Faber, Steffen Heegaard, Karine Madsen, Susanne Rosthøj, Jens Folke Kiilgaard

**Affiliations:** 1 Department of Ophthalmology, Copenhagen University Hospital Rigshospitalet, Copenhagen Ø, Denmark; 2 Department of Radiology, Copenhagen University Hospital Rigshospitalet, Copenhagen Ø, Denmark; 3 Department of Pathology, Copenhagen University Hospital Rigshospitalet, Copenhagen Ø, Denmark; 4 Department of Clinical Physiology and Nuclear Medicine, Copenhagen University Hospital Rigshospitalet, Copenhagen Ø, Denmark; 5 Statistics and Data Analysis, The Danish Cancer Society, Copenhagen Ø, Denmark

**Keywords:** uveal melanoma, metastasis, prognostic model, validation, calibration, discrimination

## Abstract

**Purpose:**

The purpose of this study was to validate the four prognostic models in patients with metastatic posterior uveal melanoma: (I) the American Joint Committee on Cancer (AJCC) staging system, (II) the Helsinki University Hospital Working Formulation (HUHWF), and two nomograms (III) the Valpione-nomogram, and (IV) the Mariani-nomogram.

**Methods:**

One hundred fifty-two patients with metastatic posterior uveal melanoma were retrospectively included. Dictated by data availability, five subcohorts were established: AJCC (*n* = 152), HUHWF (*n* = 93), Valpione-nomogram (*n* = 92), Mariani-nomogram (*n* = 68), and a complete dataset subcohort (*n* = 64). The predictive performance was evaluated with time-dependent Brier-score, calibration plots, receiver operating characteristic (ROC) curves, and the global Harrell's C-index.

**Results:**

The 6-month area under the ROC curve (AUC) was between 0.83 and 0.87 for, respectively, the Mariani-nomogram, the HUHWF, and the Valpione-nomogram, and was 0.77 for the AJCC staging system. The 24-month AUC was 0.81 for the Mariani-nomogram, compared with 0.79 (Valpione-nomogram), 0.74 (HUHWF), and 0.64 (AJCC). The C-index was 0.69 for the Mariani-nomogram, 0.71 for the Valpione-nomogram, and 0.73 for HUHWF (not calculated for AJCC). The accuracy of the prediction models, represented by the Brier score, was after 6 months and 24 months: 0.08 and 0.13 for the Mariani-nomogram, 0.10 and 0.17 for the Valpione-nomogram, 0.10 and 0.14 for the HUHWF, and 0.15 and 0.14 for the AJCC staging system.

**Conclusions:**

All four models demonstrated an acceptable predictive performance at 6 and 24 months. The Mariani-nomogram appears to perform well across most metrics, often showing the highest values for AUC and the lowest Brier scores.

##  

Posterior uveal melanoma (PUM; here defined as melanoma in the non-ultraviolet exposed area of the uvea: the choroid and the ciliary body) is a rare melanoma subtype.^1^ The worldwide incidence rate is reported to vary from about 1 and up to 11 cases per million person-years.^2^^,^^3^ PUM has a high propensity for developing metastatic spread, and about half of the patients die due to distant metastases.^4^ Once distant metastases are diagnosed, survival chances decrease markedly, with a median survival of about 1 year.^5^^,^^6^ The poor prognosis is primarily caused by the limited availability of effective treatment options, as immune checkpoint inhibitors have limited effect in patients with metastatic PUM.^7^ The conduction of randomized treatment studies is challenged by the rarity of metastatic PUM and its heterogeneous clinical course. Although the median overall survival for patients with metastatic PUM is short, patients exhibit varying survival times, with some patients surviving more than 2 years or longer with metastatic disease.^8^^–^^10^ This clinical heterogeneity emphasizes the need for accurate prognostication and proper stratification of patients when conducting clinical trials.

During the past decades, there has been a growing focus on prognostication of patients with metastases from PUM, and various prognostic factors have been identified, for example, the number of metastatic liver lesions, the performance status, the disease-free interval (DFI), liver enzyme levels, and the largest diameter of the largest metastatic lesion (LDLM).^11^^–^^15^ The LDLM has been included in the eighth edition of the American Joint Committee on Cancer (AJCC) staging manual.^16^ The AJCC is based on Tumor, Node, and Metastasis (TNM) classification, and patients with metastatic PUM are staged into three M1 categories, depending on the size of the LDLM: LDLM < 3 cm (M1a), LDLM 3.1 to 8.0 cm (M1b), and LDLM > 8.0 cm (M1c). The AJCC M1 classification allows for the stratification of patients with metastatic PUM. There are three other prognostic models for survival prediction in patients with metastatic PUM: the Helsinki University Hospital Working Formulation (HUHWF)^11^^,^^12^^,^^17^ and two nomograms: one proposed by Valpione et al. (Valpione-nomogram),^13^ and one proposed by Mariani et al. (Mariani-nomogram).^14^ The HUHWF and the two nomograms are multifactor models, accounting for several prognostic factors that will be presented in the following. This study evaluated the prognostic value of these four prognostic models in a retrospective cohort of Danish patients with metastatic PUM.

### Definition of Subcohorts

The four prognostic models each include distinct prognostic parameters (Table 1). A proxy for the tumor size/tumor burden is included in all four models: (1) the AJCC and HUHWF include the LDLM measured on computed tomography (CT), magnetic resonance imaging (MRI), or liver ultrasonography; (2) the Valpione-nomogram includes the percentage of metastatic tissue in the liver measured on CT or MRI; and (3) the Mariani-nomogram includes both the number of metastatic lesions and the surface area of the largest metastatic lesion measured in two dimensions. The liver function is included as alkaline phosphatase (ALP) in the HUHWF, whereas the cellular turnover is reflected as lactase dehydrogenase (LDH) in the Valpione-nomogram and the Mariani-nomogram. The performance status (either Eastern Cooperative Oncology Group [ECOG] or Karnofsky), which reflects a patient's overall functional ability and general health, is included in two models (the HUHWF and the Valpione-nomogram). The DFI is included in two models (the Valpione-nomogram and the Mariani-nomogram), where a short DFI is associated with shorter survival.

**Table 1.** Prognostic Factors for Survival in Metastatic Uveal Melanoma

	AJCC	Helsinki University Hospital Working Formulation	Valpione-Nomogram	Mariani-Nomogram
The largest diameter of the largest metastatic lesion, mm	+	+	–	–
Performance status	–	+	+	–
ALP level	–	+	–	–
LDH level	–	–	+	+
Liver substitution, %	–	–	+	–
Disease-free interval	–	–	+	+
Area of the biggest liver metastasis on MRI mm^2^	–	–	–	+
Number of liver metastases on MRI	–	–	–	+

AJCC, American Joint Committee on Cancer; ALP, alkaline phosphatase; LDH, lactate dehydrogenase; LDLM, largest diameter of the largest metastatic lesion; mm^2^, cubic millimeter.

## Methods

### Patients

Two hundred fifty-two Danish patients with metastatic PUM (ciliary body or choroidal melanoma) with primary PUM diagnosed from January 1, 2000, to December 31, 2020, and with development of PUM metastases until December 31, 2022 were identified in the Copenhagen Epidemiological Uveal Melanoma Study (COEUS) database.^18^ Of these, three patients were excluded due to the simultaneous presence of other cancer diseases.

Based on the prognostic factors, five subcohorts were established: (1) the AJCC subcohort, including all patients of whom it was possible to retrieve LDLM; (2) the HUHWF subcohort, including all patients with data on LDLM, performance status, and ALP level; (3) the Valpione subcohort, including all patients with data on DFI, LDH, performance status, and percentage of metastatic tissue in the liver; (4) the Mariani subcohort, including all patients with data on DFI, LDH, the surface area of the largest liver metastasis in mm^2^, and the number of liver metastases on MRI; and (5) a complete dataset subcohort (subanalysis), including patients with all the data required for all four models. Eligible patients were included in the five subcohorts defined above if a complete dataset specific to that model was available. Patients were excluded if missing data hindered scoring according to any prognostic model. As a result, the subcohort sizes varied for each model, depending on data availability for the relevant prognostic factors, and some patients were represented in all five subcohorts. The included patients were a subset of a previously published cohort.^6^

### Data Collection

The data were collected from the COEUS database or the medical records. The following data at the time of metastatic diagnosis were collected: age, sex, LDLM of any organ (measured on liver ultrasonography, MRI, CT, or positron emission tomography [PET]-CT, and was either retrieved from imaging reports or measured directly on the CT or liver MRI by two experienced radiologists), AJCC M1 stage, ECOG performance status, LDH level, ALP level, percentage of liver infiltrated by metastatic tissue, area of the largest metastatic liver lesion on MRI, and the number of liver metastases on MRI. The percentage of liver infiltrated by metastatic tissue was evaluated visually on a CT scan by one experienced radiologist or on an MRI scan by another experienced radiologist. All MRI scans from patients included in the Mariani-nomogram cohort were reviewed by one experienced radiologist, who measured the area of the largest metastatic liver lesion and the number of metastases. The DFI (calculated as the interval from the date of diagnosis of the primary posterior uveal melanoma to the date of first radiologically verified metastatic lesion), and overall survival (OS) calculated as the interval from the date of metastatic diagnosis until either the date of death of any cause or date of the last follow-up. Death by metastatic PUM was the cause of death in all patients; hence, overall death was equal to cause-specific death. Vital status was updated until December 31, 2023 (the end of the follow-up). Nine patients were still alive in the AJCC subcohort, secen in the HUHWF and the Valpione-nomogram subcohorts, and six in the Mariani-nomogram subcohort at the end of the follow-up. The study followed the Declaration of Helsinki, and the regional ethics committee approved the study with a waiver of informed consent (reference number: H-21015415).

### Calculation of Predicted Survival According to the Four Prognostic Models

#### AJCC Staging System Eighth Edition

The predicted 6-month, 12-month, 18-month, and 24-month survival probabilities for the AJCC M1 categories were obtained from the Kaplan-Meier curve “Fig. 67.3” in the AJCC staging manual, Eighth Edition.^16^

#### Helsinki University Hospital Working Formulation

Patients were staged into the IVa, IVb, and IVc HUHWF categories using the online HUHWF prognostication calculator (http://www.prognomics.org/huhwf.aspx [accessed January 16, 2025]). The predicted 6-month, 12-month, 18-month, and 24-month survival probabilities were obtained from the Kaplan-Meier curve by Kivelä et al.^12^ The individual predicted median survival in months for each patient was calculated using the online HUHWF prognosticator available at Ref. 19.

#### Valpione-Nomogram and Mariani-Nomogram

The Valpione-nomogram is available in Valpione et al.,^13^ and the Mariani-nomogram is available in Mariani et al.^14^ The predicted survival probabilities were obtained similarly for both nomograms. Each patient was evaluated based on the four prognostic factors included in the respective nomogram (see Table 1)Z. For each prognostic factor, the patients were assigned a score according to its corresponding scale on the nomogram. The total points were summed, and the predicted survival probabilities at 6 months, 12 months, and 24 months were determined by the total score on the nomogram and then referencing the corresponding survival probabilities.

### Statistics

Statistical calculations were conducted in R software (version 4.3.0).^20^ The primary endpoint was OS from when the first metastatic lesion was detected on any imaging modality until the patient's death or last follow-up. The patients alive at the end of follow-up were censored. The performance of the four prognostic models was evaluated and compared using measures of discrimination and calibration. For all measures, 95% confidence intervals (CIs) were calculated using the bootstrap method with 1000 bootstrap samples. To evaluate the discriminative performance, Harrell's C-index (referred to as C-index) was calculated.^21^ The C-index was calculated for the Valpione-nomogram, the Mariani-nomogram, and the HUHWF using the individual survival estimates. We did not calculate the C-index for AJCC because the discriminative power and interpretability are limited when applied to prognostic models with only a few distinct risk categories.

Time-dependent receiver operating characteristic (ROC) curves with the corresponding area under the curve (AUC) were also calculated to evaluate the discriminative performance at specific time points. There is no consensus definition of an acceptable C-index or AUC level. Still, a C-index or an AUC under 0.5 is considered to indicate no discriminatory ability, values between 0.6 and 0.7 indicate some discriminative ability, 0.7 to 0.8 = acceptable discriminative performance, 0.8 to 0.9 = excellent discriminative ability, and values above 0.9 = outstanding discriminative ability.^22^^,^^23^

Time-dependent Brier Scores were calculated to evaluate the four models’ overall performance, simultaneously measuring their discrimination and calibration at various time points. Lower scores indicate better predictive accuracy with 0 indicating perfect accuracy.^24^^,^^25^

Time-dependent calibration plots were generated to visually compare the observed and predicted survival probabilities across the AJCC and HUHWF staging categories. To perform calibration plots for the two nomograms, patients were divided into three groups using the tertiles of the survival probabilities from the Valpione-nomogram and the Mariani-nomogram. In a perfectly calibrated model, the points will fall precisely on the diagonal line, indicating that the predicted and observed survivals are the same.

As all data required for all four prognostic models were not available for all patients, we performed a subanalysis including only patients with all relevant data available to allow for a direct comparison of the measures.

## Results

**Figure 1.**
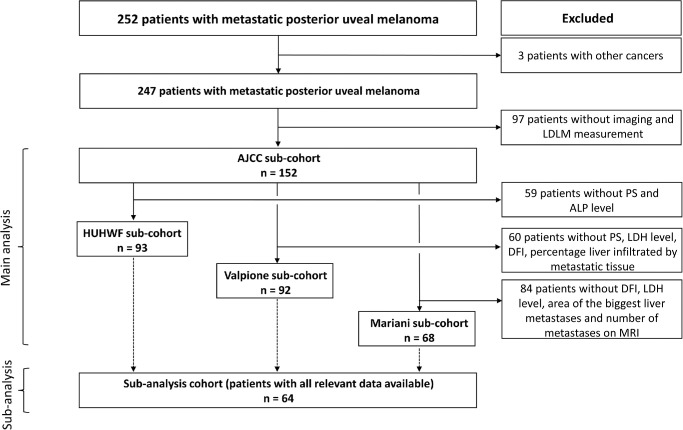
Flow chart showing the process of inclusion and exclusion of patients into the validation cohorts for each of the four prognostic models. AJCC, American Joint Committee on Cancer; ALP, alkaline phosphatase; DFI, disease-free interval; HUHWF, Helsinki University Hospital Working Formulation; LDH, lactate dehydrogenase; LDLM, largest diameter of the largest metastatic lesions; MRI, magnetic resonance imaging; PS, performance status.

**Figure 2.**
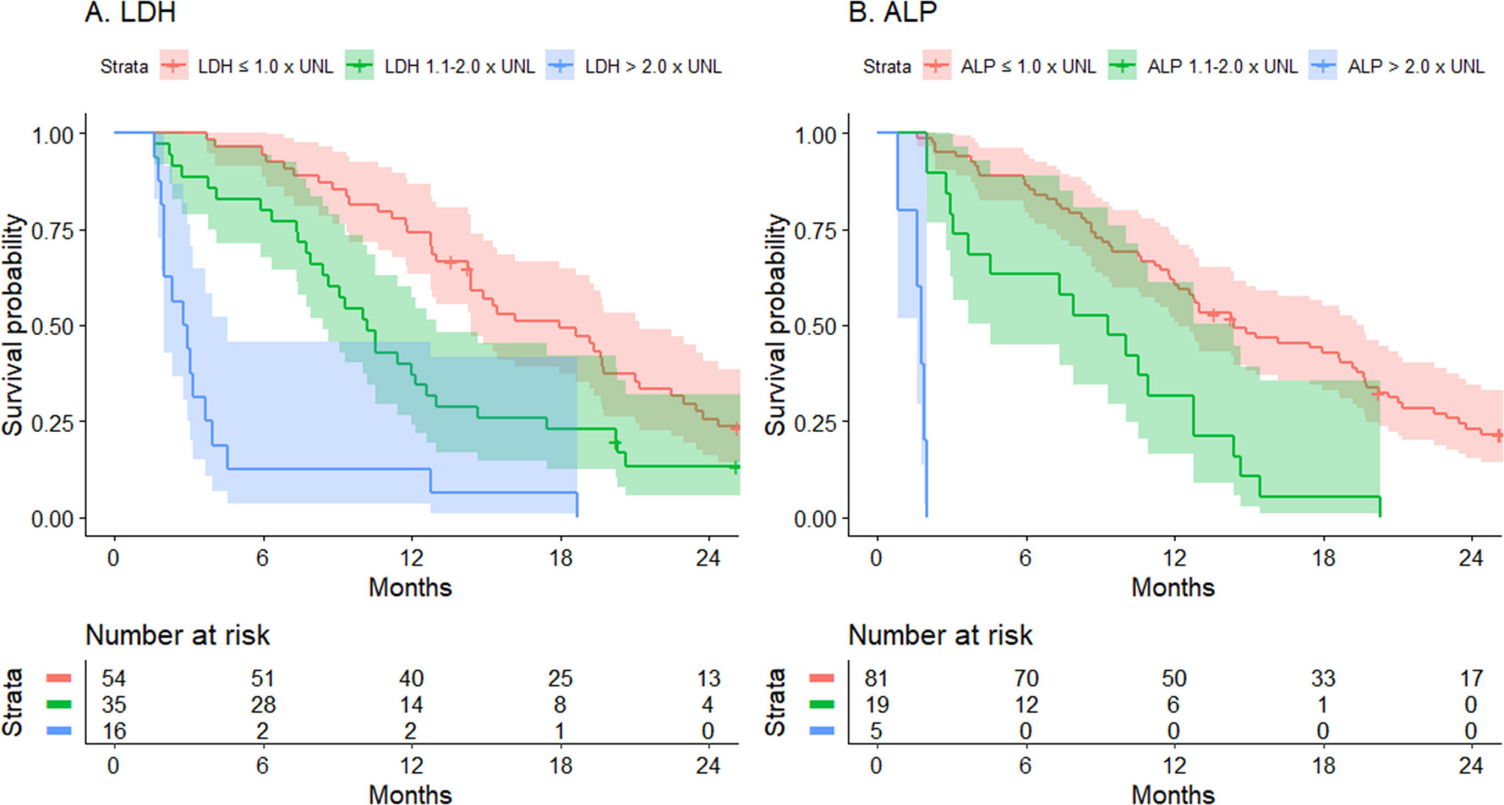
Kaplan-Meier curves of overall survival from the date of metastatic diagnosis, stratified by (**A**) lactase dehydrogenase level (LDH) levels as the fraction of upper normal limit (UNL; *n* = 105), and (**B**) alkaline phosphatase level (ALP) as a function of upper normal limit (UNL; *n* = 103). Increasing LDH and ALP was associated with poorer survival outcomes (pair-wise log-rank test: LDH ≤ 1.0 × UNL versus LDH 1.1-2-0 × UNL [*P* = 0.023], LDH ≤ 1.0 × UNL versus LDH < 2.0 × UNL [*P* < 0.001], LDH 1.1-2-0 × UNL versus LDH < 2.0 × UNL [*P* < 0.001], ALP ≤ 1.0 × UNL versus ALP 1.1-2-0 × UNL [*P* = 0.023], ALP ≤ 1.0 × UNL versus ALP < 2.0 × UNL [*P* < 0.001], and ALP 1.1-2-0 × UNL versus ALP < 2.0 × UNL [*P* < 0.001]). ALP, alkaline phosphatase; LDH, lactate dehydrogenase; UNL, upper normal limit.

From the cohort of 252 patients, the LDLM was available in 152 patients. Sixty-nine patients were diagnosed with metastatic disease before 2009, of which 62 patients were excluded (90%) due to missing data on LDLM, whereas 183 patients were diagnosed with metastatic disease after 2009, of which 38 patients were excluded (20%). Patients with LDLM measurements were included in the AJCC subcohort (main analysis) (Fig. 1). From the AJCC subcohort, 93 patients were included in the HUHWF subcohort (main analysis), 91 in the Valpione subcohort (main analysis), 68 in the Mariani subcohort (main analysis), and 64 in the subcohort with a complete dataset (subanalysis; see Fig. 1). Patient characteristics are presented in Table 2. In total, the LDH level was available in 105 patients and the ALP level in 103 patients. Kaplan-Meier curves of the OS probabilities stratified by LDH and ALP levels are presented in Figure 2.

**Table 2.** Patient Characteristics for Each Prognostic Model Cohort

	AJCC (Total Cohort) *n* = 152	Helsinki University Hospital Working Formulation *n* = 93	Valpione-Nomogram *n* = 92	Mariani-Nomogram *n* = 68	Complete Dataset Subcohort
Sex					
Women	82 (54%)	53 (57%)	51 (55%)	37 (54%)	35 (55%)
Men	70 (46%)	40 (43%)	41 (45%)	31 (46%)	29 (45%)
Age at metastatic diagnosis					
Median, y	66 (IQR = 59–74)	65 (IQR = 58–74)	65 (IQR = 58–73)	65 (IQR = 57–72)	65 (IQR = 57–72)
≤ 60 y of age	39 (26%)	27 (29%)	28 (30%)	20 (29%)	20 (31%)
> 60 y of age	113 (74%)	66 (71%)	64 (70%)	48 (71%)	44 (69%)
Largest diameter of the largest metastatic lesion					
Median, mm	34 (IQR = 20–60)	30 (IQR = 18–50)	30 (IQR = 18–52)	25 (IQR = 18–42)	23 (17–42)
AJCC stage					
M1a	68 (45%)	48 (51%)	47 (51%)	39 (57%)	37 (58%)
M1b	59 (39%)	37 (40%)	36 (39%)	23 (34%)	22 (34%)
M1c	25 (16%)	8 (9%)	9 (10%)	6 (9%)	5 (8%)
Performance status (WHO/ECOG)					
PS 0–1	107 (70%)	79 (85%)	78 (85%)	58 (85%)	61 (95%)
PS 2–4	15 (11%)	14 (15%)	14 (15%)	10 (15%)	3 (5%)
Missing value	29 (19%)	0	0	0	
Lactic dehydrogenase					
1.0 × UNL	54 (36%)	47 (50%)	48 (52%)	41 (60%)	40 (63%)
1.1 – 1.5 × UNL	28 (18%)	24 (26%)	23 (25%)	16 (24%)	15 (23%)
> 1.5 × UNL	23 (15%)	21 (23%)	21 (23%)	11 (16%)	9 (14%)
Missing value	47 (31%)	1 (1%)	0	0	
Alkaline phosphatase					
1.0 × UNL	81 (53%)	71 (76%)	69 (75%)	58 (85%)	55 (86%)
1.1 – 1.5 × UNL	18 (12%)	18 (20%)	16 (17%)	9 (13%)	9 (14%)
> 1.5 × UNL	6 (4%)	4 (4%)	5 (6%)	1 (2%)	0 (0%)
Missing value	47 (31%)	0	2 (2%)	0	
Percentage metastatic liver					
0%	7 (5%)	7 (8%)	7 (8%)	0	0
< 20%	63 (41%)	60 (65%)	63 (68%)	53 (78%)	53 (83%)
20–50%	15 (10%)	14 (15%)	15 (16%)	11 (16%)	10 (16%)
> 50%	7 (5%)	6 (6%)	7 (8%)	2 (3%)	1 (1%)
Missing value	60 (39%)	6 (6%)	0	2 (3%)	
Area of the largest liver metastasis (mm^2^)					
1–500	31 (20%)	31 (33%)	30 (33%)	31 (46%)	30 (47%)
501–800	5 (3%)	4 (4%)	4 (4%)	5 (7%)	4 (6%)
801–1200	10 (7%)	10 (11%)	10 (11%)	10 (15%)	10 (16%)
> 1200	22 (15%)	20 (22%)	22 (24%)	22 (32%)	20 (31%)
Missing value	84 (55%)	28 (30%)	26 (28%)		
Number of metastatic lesions on liver					
1–4	21 (14%)	20 (21%)	19 (21%)	21 (31%)	19 (30%)
5–10	10 (7%)	10 (11%)	10 (11%)	10 (15%)	10 (16%)
> 10	37 (24%)	35 (38%)	37 (40%)	37 (54%)	35 (54%)
Missing value	84 (55%)	28 (30%)	26 (28%)	0	
Disease-free interval					
Median, mo	28.0 (IQR = 12.0–56.0)	25.0 (IQR = 11.0–41.0)	25.0 (IQR = 11.0–43.0)	26.5 (IQR = 11.0–37.0)	25.0 (IQR = 11.0–36.0)
Disease-free interval groups					
0–6 mo	21 (14%)	13 (14%)	12 (13%)	10 (15%)	9 (14%)
7–12 mo	20 (13%)	14 (15%)	16 (17%)	10 (15%)	10 (16%)
13–24 mo	25 (16%)	18 (19%)	17 (19%)	12 (17%)	12 (19%)
> 25 mo	86 (57%)	48 (52%)	47 (51%)	36 (53%)	33 (51%)
Overall survival					
Median, mo	11.2 (95% CI = 9.36–12.8)	12.8 (95% CI = 11.4–16.2)	12.8 (95% CI = 11.4–16.2)	14.7 (95% CI = 12.8–19.7)	14.9 (95% CI = 13.0–19.7)

The AJCC cohort represent the total cohort.

AJCC, American Joint Committee on Cancer; ECOG, Eastern Cooperative Oncology Group; IQR, interquartile range; mm^2^, square millimeter; PS, performance status; UNL, upper normal limit; WHO, World Health Organization.

### Brier Score Assessment (Overall Performance)

Brier scores of the main analyses and subanalysis are presented in Table 3. In the subanalysis on patients with complete data, the Brier scores of the four models are comparable across the timeframes with overlapping CIs. The Mariani-nomogram demonstrated the lowest Brier score at 24 months in both the main analysis (0.13, 95% CI = 0.08–0.19) and the subanalysis (0.14, 95% CI = 0.08–0.20). However, the CIs overlap with the CIs of the 24-month Brier scores for the other three models.

**Table 3.** Time-Dependent Brier Score, Time-Dependent ROC AUC, and Harrell's C-Index for all Four Prognostic Models

	American Joint Committee on Cancer n = 152 (95% CI)	Helsinki University Hospital Working Formulation *n* = 93 (95% CI)	Valpione-Nomogram *n* = 92 (95% CI)	Mariani-Nomogram *n* = 68 (95% CI)
Main analysis				
No. of patients	152	93	92	68
Overall performance				
Brier score				
6 mo	0.15 (0.12–0.19)	0.10 (0.06–0.15)	0.10 (0.06–0.16)	0.08 (0.05–0.14)
12 mo	0.22 (0.19–0.26)	0.18 (0.15–0.22)	0.20 (0.17–0.26)	0.19 (0.15–0.26)
18 mo	0.19 (0.17–0.22)	0.18 (0.17–0.21)	–	–
24 mo	0.14 (0.11–0.18)	0.14 (0.11–0.17)	0.17 (0.14–0.20)	0.13 (0.08–0.19)
Discriminative performance				
ROC AUC				
6 mo	0.77 (0.66–0.90)	0.84 (0.72–0.93)	0.87 (0.76–0.96)	0.83 (0.64–0.98)
12 mo	0.70 (0.62–0.81)	0.80 (0.71–0.88)	0.79 (0.70–0.88)	0.79 (0.67–0.89)
18 mo	0.70 (0.62–0.80)	0.77 (0.70–0.85)	–	–
24 mo	0.64 (0.53–0.77)	0.74 (0.68–0.80)	0.79 (0.66–0.89)	0.81 (0.66–0.93)
Harrell's C-index	–	0.73 (0.65–0.81)	0.71 (0.64–0.80)	0.69 (0.59–0.79)
Subanalysis of patients with complete data			
No. of patients	64	64	64	64
Overall performance				
Brier score				
6 mo	0.07 (0.04–0.10)	0.06 (0.03–0.12)	0.08 (0.04–0.15)	0.07 (0.04–0.11)
12 mo	0.19 (0.16–0.25)	0.17 (0.14–0.23)	0.18 (0.14–0.25)	0.2 (0.14–0.26)
18 mo	0.22 (0.19–0.26)	0.21 (0.19–0.25)	–	–
24 mo	0.17 (0.12–0.22)	0.17 (0.13–0.21)	0.20 (0.18–0.24)	0.14 (0.08–0.20)
Discriminative performance				
ROC AUC				
6 mo	0.92 (0.82–0.99)	0.88 (0.69–0.99)	0.81 (0.58–0.97)	0.89 (0.73–0.99)
12 mo	0.71 (0.57–0.83)	0.72 (0.60–0.85)	0.78 (0.65–0.88)	0.79 (0.67–0.90)
18 mo	0.67 (0.56–0.78)	0.70 (0.61–0.79)	–	–
24 mo	0.63 (0.50–0.76)	0.65 (0.59–0.72)	0.73 (0.56–0.88)	0.80 (0.64–0.93)
Harrell's C-index	–	0.69 (0.59–0.80)	0.67 (0.55–0.78)	0.69 (0.59–0.79)

Eighteen-month survival probabilities were not available for the Valpione-nomogram and the Mariani-nomogram. The C-index was not calculated for the American Joint Committee on Cancer Staging System, as the three AJCC stages provide too few distinct risk groups to allow for reliable pairwise comparisons.

AUC, area under the curve; C-index, Concordance Index; CI, confidence interval; ROC, receiver operating characteristics curve.

### Calibration

**Figure 3.**
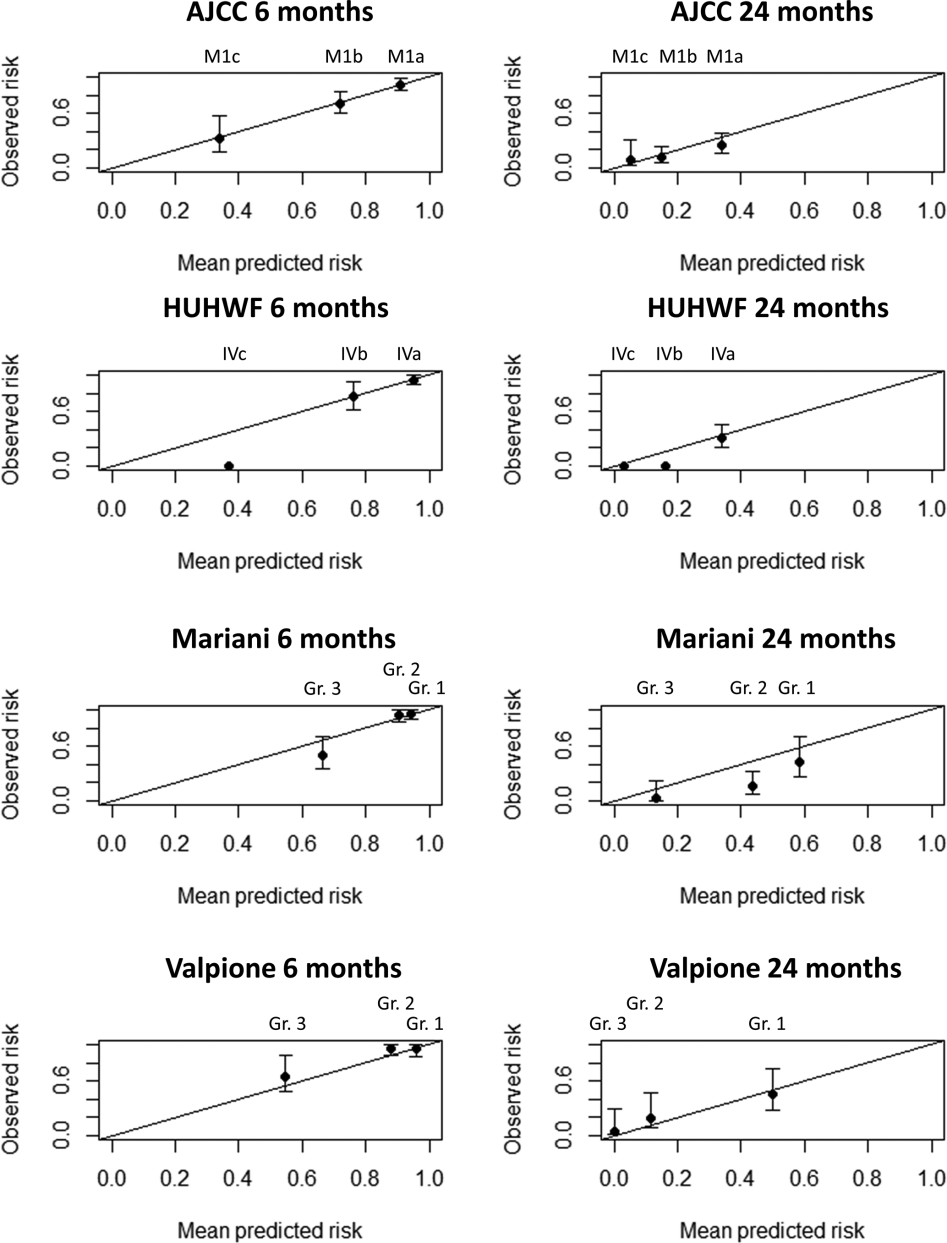
Calibration plots of predicted survival at 6 and 24 months for the four prognostic models (calculated on the main analysis subcohorts). The diagonal lines represent a perfect prognostic model. A point above the diagonal line indicates that the patients exhibit longer survival times than predicted by the model. Conversely, a point below the diagonal line indicates that the patients die earlier than the model predicts. Bars indicate the 95% confidence interval for the observed risk. AJCC, American Joint Committee on Cancer; HUHWF, Helsinki University Hospital Working Formulation.

Figure 3 presents 6-month and 24-month calibration plots for the main analysis subcohorts (12-month and 18-month calibration plots are presented in Supplementary Fig. S1). The AJCC has a nearly perfect calibration at 6 months and demonstrates quite accurate calibration at 12, 18, and 24 months. The HUHWF has a near-perfect calibration at 6 months for HUHWF stage IVa and IVb, but all patients with stage IVc have died earlier than 6 months or are censored because they were alive at the end of follow-up. HUHWF stage IVa demonstrates a good calibration across the timeframes, but at 24 months, all patients with stage IVb have died earlier than the model predicts or have been censored. To perform calibration plots for the two nomograms, patients were categorized into three groups corresponding to the tertiles (Kaplan-Meier curves of OS stratified by the Valpione-nomogram groups and the Mariani-nomogram groups are presented in Supplementary Fig. S2). For the Valpione-nomogram, patients tend to die earlier than predicted by the nomogram at 12 and 24 months (see Fig. 3). The Mariani-nomogram demonstrates a reasonably good calibration across the timeframes.

### Discriminative Performance

**Figure 4.**
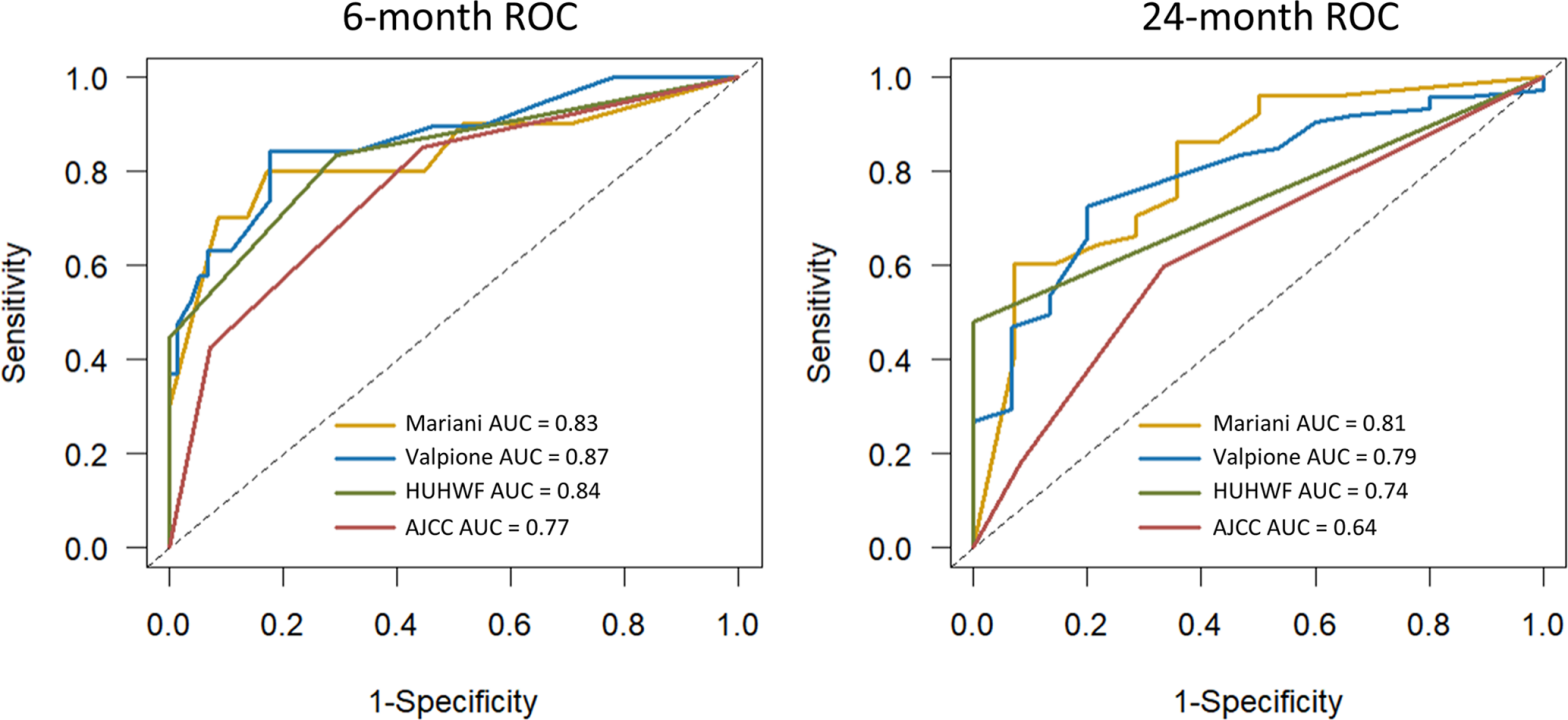
Six-month and 24-month ROC curves for the AJCC, the HUHWF, the Valpione-nomogram, and the Mariani-nomogram. AJCC, American Joint Committee on Cancer; AUC, area under the curve; HUHWF, Helsinki University Hospital Working Formulation; ROC, receiver operation characteristic.

Time-dependent ROC curves for the 6-month and 24-month predictions for the main analyses are presented in Figure 4 and the 12-month and 18-month ROC curves for the main analyses are shown in Supplementary Figure S3. The ROC AUCs from the main analyses and subanalysis are presented in Table 3. In the subanalysis, the ROC AUCs are comparable across the different timeframes with overlapping CIs. The Mariani-nomogram demonstrated the highest ROC AUC score at 24 months in both the main analysis (0.81, 95% CI = 0.66–0.93) and the subanalysis (0.80, 95% CI = 0.64–0.93; see Table 3). However, the CIs overlap with the CIs of the 24-month ROC AUCs for the other three models. The C-indexes from the main analyses and the subanalysis are presented in Table 3.

## Discussion

In this comparison of four prognostic models for the survival of patients with metastatic posterior uveal melanoma, we find all models to have a general good performance with an acceptable discriminative ability, calibration, and overall performance up to 24 months. However, our data did not enable us to identify a superior model, as the CIs were broad, leading to considerable uncertainty in the estimates. Additionally, the sizes of our subcohorts varied between model validations, making direct comparisons questionable. To address this, we conducted a subanalysis on the subset of 64 patients who met all the requirements for all models. However, this subcohort was highly selected, as only well-cooperating patients could meet all the criteria. Despite this limitation, the subanalysis showed a trend toward the Mariani-nomogram being the best model for predicting 24-month survival, a noteworthy endpoint in clinical trials. This observation aligns with the findings of the original article by Mariani et al.,^14^ in which the authors also found the best calibration at 24 months. Mariani et al.^14^ reported a C-index of 0.71 (the CI is not reported), which is comparable to the C-index of 0.69 (95% CI = 0.59-0.79) we calculated for the Mariani subcohort. A similar correlation is found for the Valpione-nomogram,^13^ where the authors report a C-index of 0.75 in their development dataset, and 0.80 in the externally validated dataset (the CIs are not reported), which are comparable to the Valpione-nomogram C-index in our study (0.71, 95% CI = 0.64-0.80).

It is, to the best of our knowledge, the first time a comparison between different prediction models has been performed on a consecutive cohort of patients with metastatic posterior uveal melanoma. The four models incorporate different prognostic factors related to various aspects of the disseminated disease, but a proxy for tumor size/tumor burden is incorporated in all four models: the LDLM, percentage of liver infiltrated by metastatic tissue, and the number and surface area of the largest liver metastasis. The LDLM can be obtained from various imaging modalities,^12^ which makes the AJCC staging system more easily applicable in clinical practice. Besides LDLM, the HUHWF includes the ALP level and the performance status,^11^ which are easily accessible at most hospitals. The online HUHWF prognosticator also allows for easy implementation of the HUHWF in clinical practice. Valpione et al. calculated the percentage of liver metastases in three dimensions on a helical CT or MRI, using a dedicated software program.^13^ In the present study, we did not have the resources to obtain the exact volume. Instead, the percentage of metastatic tissue within the liver (< 20%, 20%–50%, or > 50%) was evaluated visually by an experienced radiologist. The significant time required for accurate volumetric measurement poses a challenge to implementing the Valpione-nomogram in a clinical setting. It is noteworthy, that the Mariani-nomogram applies only to patients with liver metastases and requires a liver MRI for its use.^14^ With access to MRI scans, obtaining the number and surface area of the largest liver metastasis would be straightforward to implement in clinical practice. Patients in poor clinical condition might forgo a thorough investigation, either at their request or because systemic treatment is considered futile. We anticipate that the patients included in the Mariani subcohort and the subanalysis cohort were in a better clinical condition, as only patients with a performed liver MRI could be included. This is reflected in the longer median survival in the Mariani subcohort (14.7 months, 95% CI = 12.8–19.7) and the subanalysis cohort (14.9 months, 95% CI = 13.0–19.7), compared to, for example, the AJCC subcohort (11.2 months, 95% CI = 9.36–12.8). Thus, it is important to consider that the patients included in the sub-cohorts generally were in a better clinical condition than those who were excluded. Consequently, our findings are primarily applicable to patients who can undergo thorough diagnostic assessment.

In the three multi-factor models,^11^^,^^13^^,^^14^ Cox regression analyses identified additional parameters as independent predictors for survival from the time of metastatic diagnosis. ALP is used in HUHWF, whereas LDH is used in the Valpione-nomogram and Mariani-nomogram. ALP reflects the liver function and indicates if the liver is affected by metastases,^26^ whereas LDH is less specific and reflects tissue damage and cellular turnover.^27^ LDH is associated with tumor size and is also considered an indirect indicator of the tumor burden.^28^^,^^29^ In the Valpione-nomogram and Mariani-nomogram, the LDH level contributes the highest number of points relative to the other factors, indicating that the LDH level has a strong predictive power. In our cohort, the Kaplan-Meier curves also showed a clear separation across the LDH and ALP categories. However, the distribution of patients was skewed across the ALP categories, with most patients exhibiting ALP levels within the normal limits. The patients were more evenly distributed across the LDH groups. The DFI was included in both nomograms, where a short DFI was related to shorter OS, indicating a more aggressive tumor. However, in a previously published study by our research group, the DFI was not found to be an independent prognostic factor.^6^ Contrarily, the site of the metastatic lesions (the metastatic pattern) was found to be an independent factor for survival in the mentioned study, as patients presenting with sole extrahepatic metastases have significantly longer OS.^6^ However, none of the prognostic models evaluated in the present study consider the metastatic pattern.

It is important to note that the AJCC and the HUHWF staging categories are based on predicted survival rates and that they do not provide outcome predictions for each patient, although the online HUHWF prognosticator does provide an individual predicted median survival in months.^12^ The Valpione-nomogram and the Mariani-nomogram provide individual predictions in terms of 6-month, 12-month, and 24-month survival probabilities, but they do not provide any cutoff values to categorize patients into prognostic groups. Further, it should be noted that individual predictions could be used to guide tailored treatment, but clinicians should remain cautious when estimating predicted survival, as the estimates are inherently uncertain. The categorical models AJCC and the HUHWF are ideal for patient stratification in clinical trials, which, for instance, was done in the phase III Tebentafusp trial.^30^ Stratification of patients based on the Valpione-nomogram and the Mariani-nomogram would require a definition of cutoff values to assign patients into prognostic groups. In the present study, we chose to divide the Valpione-nomogram and the Mariani-nomogram cohorts into three groups based on the tertiles of the survival probabilities to compute calibration plots comparable to the AJCC and HUHWF, but we did not explore the best cutoff values.

A major limitation of this retrospective study is the missing data, which resulted in small cohorts and selection bias.^31^ There was a clear trend in the clinical work-up over time, with fewer assessments conducted in the earlier part of the study period (prior to 2009) compared to the period after 2009, when a more standardized clinical work-up program was implemented. Still, some patients declined or were unable to undergo a complete work-up, for example, claustrophobia and an MRI. Another limitation is the visual evaluation of the percentage of liver infiltrated by metastatic tissue, as opposed to the volumetric calculation in Valpione et al.^13^ Even though the percentage of liver metastases was evaluated visually in our study, the Valpione-nomogram did perform well in all analyses and was comparable to the other models. It should be noted that we included the LDLM of any metastatic lesion, although it remains unclear whether LDLM as a prognostic factor applies to extrahepatic lesions.

## Conclusions

This validation study showed that the AJCC staging system, the HUHWF, the Valpione-nomogram, and the Mariani-nomogram demonstrated acceptable predictive accuracy regarding overall performance, calibration, and discriminative ability. All four models are feasible for patient prognostication, but the broad CIs indicate high uncertainty in the estimates. Our results suggest that the Mariani-nomogram might have the best ability to identify long-term survivors of more than 2 years. Thus, the Mariani-nomogram seems to be the best choice in a clinical trial setting, where long-term survival is to be investigated. The choice of model depends on the purpose, outcome timeframe, imaging and laboratory tests accessibility, and resource considerations, as some of the prognostic factors are time-consuming to obtain, in addition to the laborious process of calculating the nomogram scores and survival probabilities. Based on clinical applicability, the AJCC staging system is straightforward and cost-effective to implement.

## Supplementary Material

Supplement 1
