# Supplementary material for: Validation of Four Prognostic Models for Metastatic Posterior Uveal Melanoma in a Danish Cohort
Source: Invest Ophthalmol Vis Sci. 2025 May 27;66(5):38. doi: 10.1167/iovs.66.5.38 (PMC12124156; doi:10.1167/iovs.66.5.38)
Supplement: Supplement 1 [file iovs-66-5-38_s001.pdf]

Supplementary

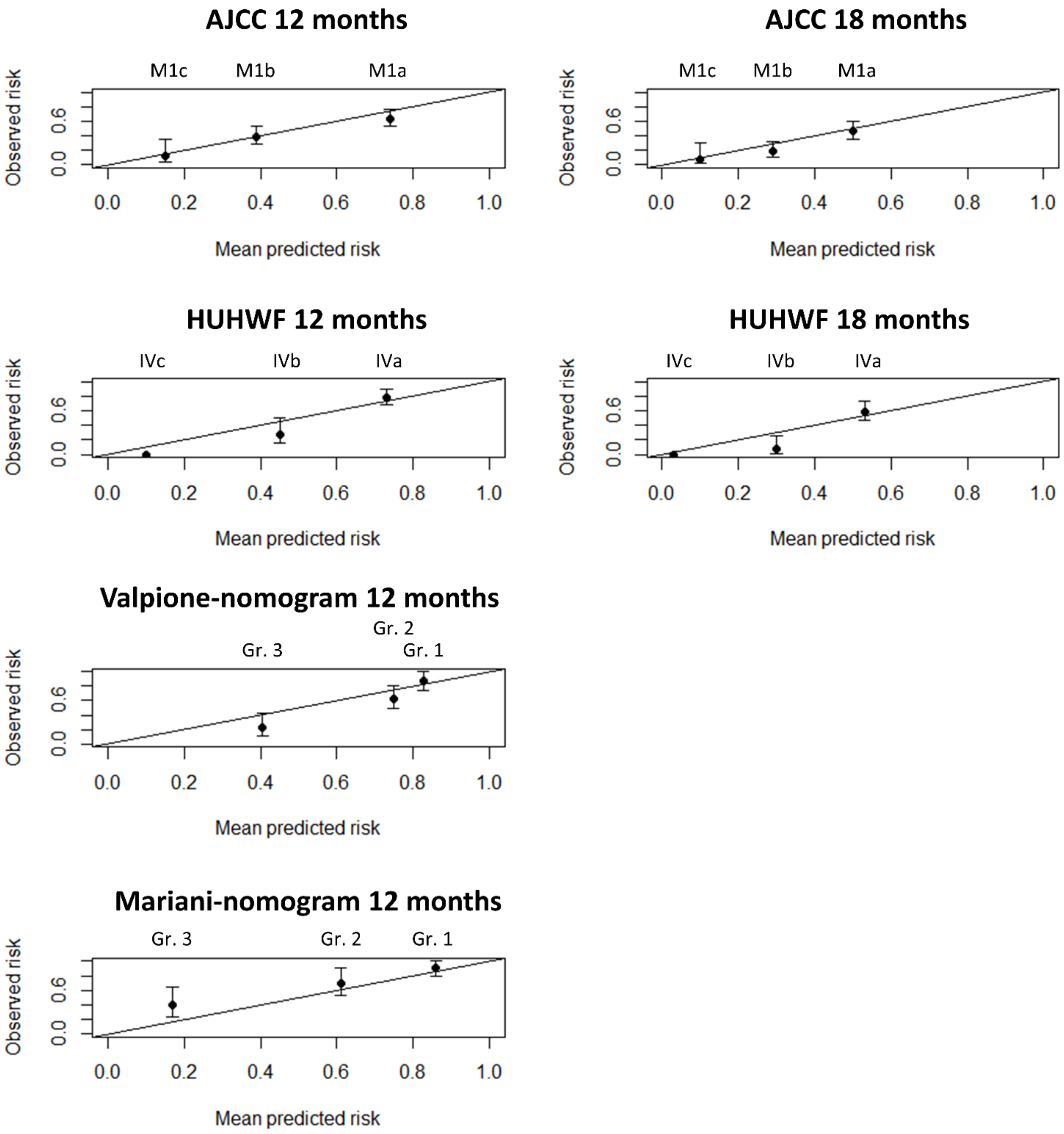

Figure S1. Calibration plots of the 12-month and 18-month predictions for the AJCC, HUHWF, Valpione-nomogram, and Mariani-nomogram cohorts (18-month predictions not available for the Valpione-nomogram and the Mariani-nomogram). Abbreviations: AJCC = American Joint Committee on Cancer, HUHWF = Helsinki University Hospital Working Formulation.

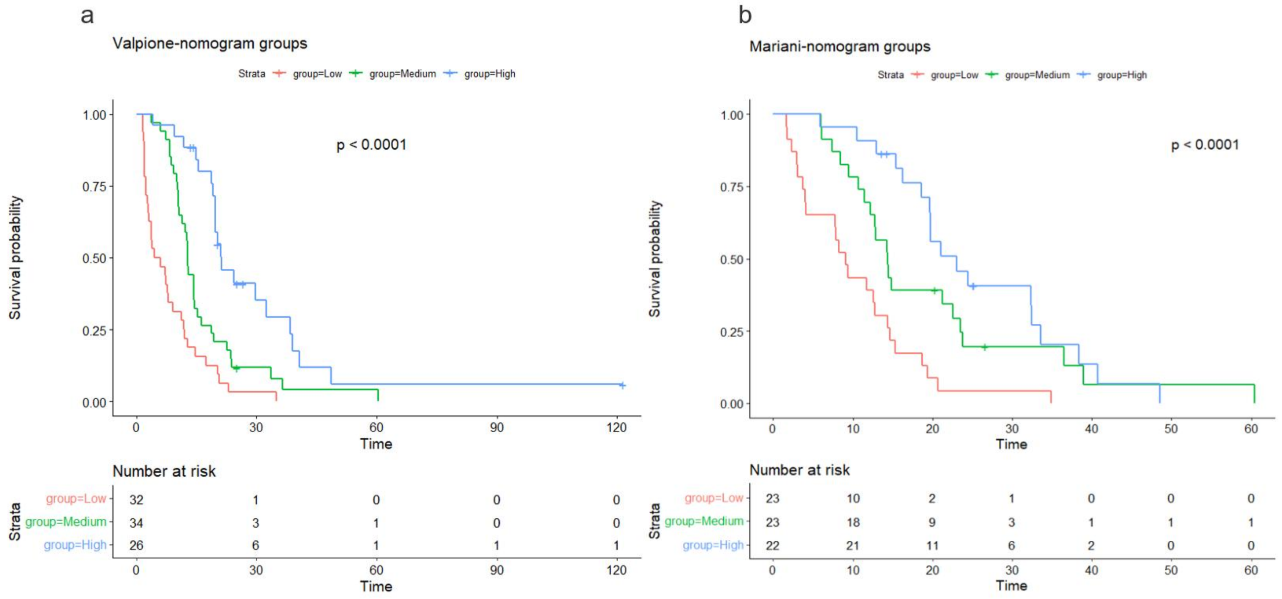

Figure S2. Kaplan-Meier plots of overall survival, stratified by the Valpione-nomogram groups (a), and Mariani-nomogram groups (b).

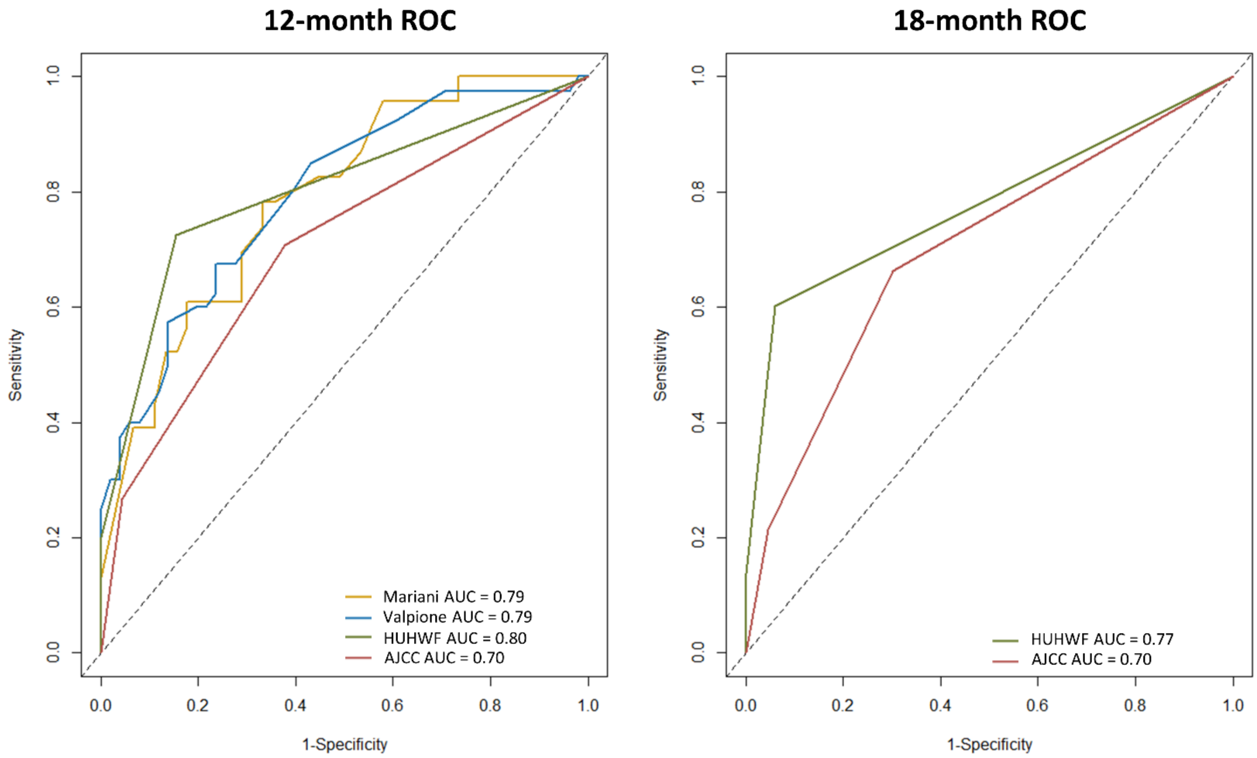

Figure S3. Twelve-month and 18-month ROC curves for the AJCC, HUHWF, Valpione-nomogram, and Mariani-nomogram sub-cohorts (18-month predictions are not available for the Valpione-nomogram and the Mariani-nomogram).
Abbreviations: AJCC = American Joint Committee on Cancer, HUHWF = Helsinki University Hospital Working Formulation, ROC = Receiver Operating Characteristics.
